# p21^Cip1^ plays a critical role in the physiological adaptation to fasting through activation of PPARα

**DOI:** 10.1038/srep34542

**Published:** 2016-10-10

**Authors:** Elena Lopez-Guadamillas, Pablo J. Fernandez-Marcos, Cristina Pantoja, Maribel Muñoz-Martin, Dolores Martínez, Gonzalo Gómez-López, Ramón Campos-Olivas, Angela M. Valverde, Manuel Serrano

**Affiliations:** 1 Tumor Suppression Group, Spanish National Cancer Research Centre (CNIO), Madrid E28029, Spain; 2 Bioactive Products and Metabolic Syndrome Group, Madrid Institute of Advanced Studies (IMDEA) in Food, CEI UAM+CSIC, Madrid E28049, Spain; 3 Flow Cytometry Unit, Spanish National Cancer Research Centre (CNIO), Madrid E28029, Spain; 4 Bioinformatics Unit, Spanish National Cancer Research Centre (CNIO), Madrid E28029, Spain; 5 Spectroscopy and Nuclear Magnetic Resonance Unit, Spanish National Cancer Research Centre (CNIO), Madrid E28029, Spain; 6 Instituto de Investigaciones Biomédicas “Alberto Sols” (CSIC/UAM), Madrid E28029, Spain; 7 Centro de Investigaciones Biomédicas en Red de Diabetes y Enfermedades Metabólicas Asociadas, ISCIII, Spain

## Abstract

Fasting is a physiological stress that elicits well-known metabolic adaptations, however, little is known about the role of stress-responsive tumor suppressors in fasting. Here, we have examined the expression of several tumor suppressors upon fasting in mice. Interestingly, *p21* mRNA is uniquely induced in all the tissues tested, particularly in liver and muscle (>10 fold), and this upregulation is independent of p53. Remarkably, in contrast to wild-type mice, *p21*-null mice become severely morbid after prolonged fasting. The defective adaptation to fasting of *p21*-null mice is associated to elevated energy expenditure, accelerated depletion of fat stores, and premature activation of protein catabolism in the muscle. Analysis of the liver transcriptome and cell-based assays revealed that the absence of p21 partially impairs the transcriptional program of PPARα, a key regulator of fasting metabolism. Finally, treatment of *p21*-null mice with a PPARα agonist substantially protects them from their accelerated loss of fat upon fasting. We conclude that p21 plays a relevant role in fasting adaptation through the positive regulation of PPARα.

Fasting has been thoroughly studied regarding the metabolic adaptations that it triggers. These adaptions basically consist in the sequential mobilization of internal nutrient stores, starting with hepatic glycogen, then triglycerides from the adipose tissue, and finally proteins from muscle^1^. However, the implications of fasting adaptation go further beyond metabolism. For example, fasting strongly diminishes the toxic effects of chemotherapy on normal cells and tissues, both in mice^2^ and humans^3^, and this has substantially increased the interest in understanding the link between stress responses and fasting.

Many tumor suppressors participate in stress signaling pathways, and this has been extensively studied in the context of cellular damage conducive to cancer, such as DNA damage and oncogenic stress^4^. However, the possible role of tumor suppressors in response to fasting has remained largely unexplored. Previous researchers have identified p21 (also known as p21^Cip1^ or CDKN1A) as a fasting-induced factor^5^, but nothing was known about the relevance and role of p21 in fasting adaptation.

The best understood functions of p21 are inhibition of the cyclin dependent kinases (primarily CDK2) and inhibition of the DNA replication processivity factor PCNA^6^, thereby efficiently blocking cell proliferation. In addition to this, p21 has a growing list of interacting partners including the binding and inhibition of transcription factors, such as E2F1, STAT3 and MYC, all of them involved in cell proliferation and survival^6^. The levels of p21 are regulated transcriptionally by p53, as well as by important negative regulators of proliferation, such as TGFβ-activated SMAD complexes and FOXO proteins^6^.

In this work, we report that p21 is a critical factor for fasting adaptation and we present evidence for an unprecedented role of p21 as a positive regulator of PPARα, a key transcription factor that orchestrates multiple aspects of fasting adaptation, including fatty acid oxidation and ketogenesis^7^,^8^,^9^.

## Results

### Characterization of fasting-induced *p21* upregulation

**Figure 1:**
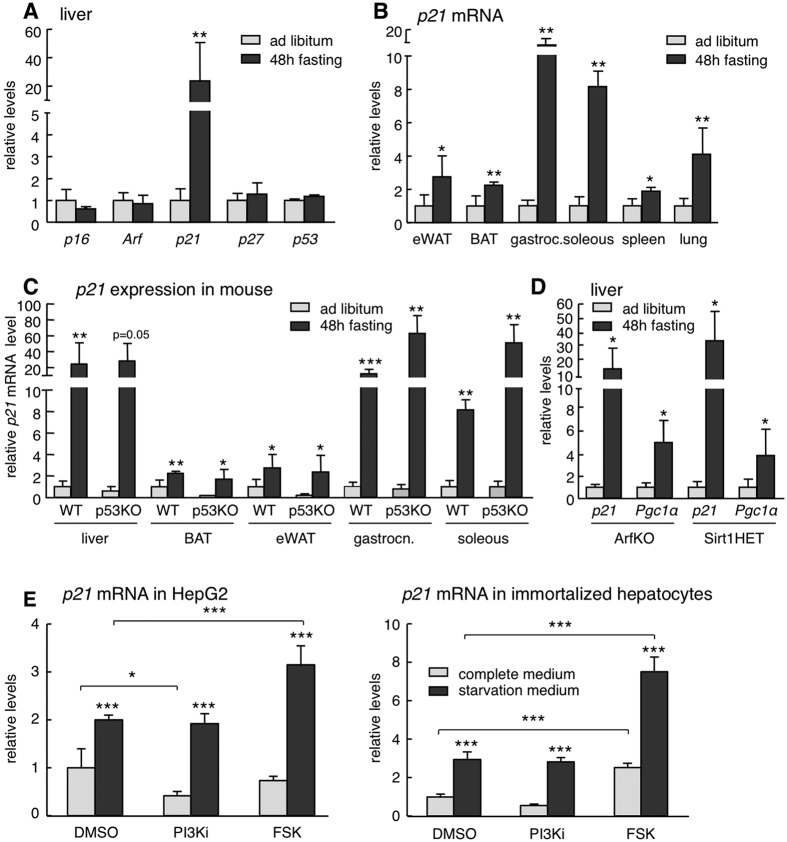
Characterization of fasting-induced *p21* upregulation. (**A**) Relative mRNA expression of the indicated tumor suppressor genes in the liver after 48 h fasting compared to ad libitum fed mice (n = 4 per group, males, 12 weeks old). (**B**) Relative *p21* mRNA levels in epididymal white adipose tissue (eWAT), brown adipose tissue (BAT), gastrocnemius, soleous, spleen and lung of mice after 48 h fasting or ad libitum feeding (same mice as in panel A). (**C**) Relative *p21* mRNA levels in the indicated tissues of ad libitum fed and 48 h fasted wild type (WT) or p53KO mice (n = 4 per group, males, 12 weeks old). (**D**) Relative *p21* and *Pgc1α* expression in the liver of ArfKO and Sirt1HET mice after 48 hours fasting or ad libitum feeding (n = 4 per group, males, 12 weeks old). The fasting-induced gene *Pgc1α* was used as control. (**E**) Relative *p21* mRNA levels in HepG2 (human hepatocellular carcinoma) cells (left) or primary large-T immortalized mouse hepatocytes (right) cultured for 24 h in complete medium (DMEM + 10%FBS) or starvation medium (glucose-free and serum-free DMEM) and treated for 5 h with DMSO, 10 μM CNIO PI3K inhibitor (PI3Ki) or 10 μM forskolin (FSK). The experiment was performed twice with similar results and each time in three biological replicates. Data correspond to one experiment (n = 3). Levels of mRNA were normalized to *β-actin*, except for muscle that is normalized to *Gapdh*. Values correspond to average ± s.d. Statistical significance was determined by the unpaired two-tailed Student’s t test (panels A to D) or by two-way ANOVA and Bonferroni post-hoc test (panel E): *p < 0.05, **p < 0.01, ***p < 0.001.

Previous investigators have reported that *p21* mRNA is upregulated in many tissues upon fasting through a mechanism that is independent of p53 and partly mediated by FOXO1^5^. We wanted to confirm and extend these findings and, for this, we began by analyzing whether other cell cycle inhibitors and tumor suppressors are also upregulated by fasting. Interestingly, only *p21* mRNA was strongly induced upon fasting in the liver, while *p16*^*Ink4a*^, *p19*^*Arf*^, *p27*^*Kip1*^, or *p53* mRNAs were unaffected (Fig. 1A). We found that this upregulation is present across all tissues tested (Fig. 1B), being more prominent in the liver and muscle. Also, fasting-induced *p21* upregulation was independent of p53 (Fig. 1C) and p19^Arf^ (Fig. 1D). The sirtuin SIRT1 is involved in many responses to nutrient deprivation^10^, but *p21* upregulation by fasting was not affected in *Sirt1*-heterozygous mice (Fig. 1D).

The induction of *p21* by nutrient deprivation was also recapitulated in cultured cells. In particular, we observed that *p21* was significantly induced when human hepatocarcinoma HepG2 cells or mouse immortalized primary hepatocytes were starved (no serum and no glucose, for 24 h) (Fig. 1E). Nutrient deprivation, among many other effects, decreases PI3K activity and elevates cAMP, both being important for metabolic adaptation^11^. To explore the contribution of these pathways to the upregulation of *p21*, cells were treated with a pharmacological inhibitor of PI3K or with forskolin (to increase cAMP levels). Interestingly, forskolin, but not PI3K inhibition, further enhanced the upregulation of *p21* by nutrient deprivation in cells (Fig. 1E). These observations suggest that cAMP, possibly through the transcription factor CREB, is involved in the upregulation of *p21* upon starvation. Together, these observations reinforce the concept that p21^Cip1^ is induced by nutrient deprivation both at the organism level and in isolated cells.

### Impaired adaptation to prolonged fasting in p21^Cip1^ deficient mice

**Figure 2:**
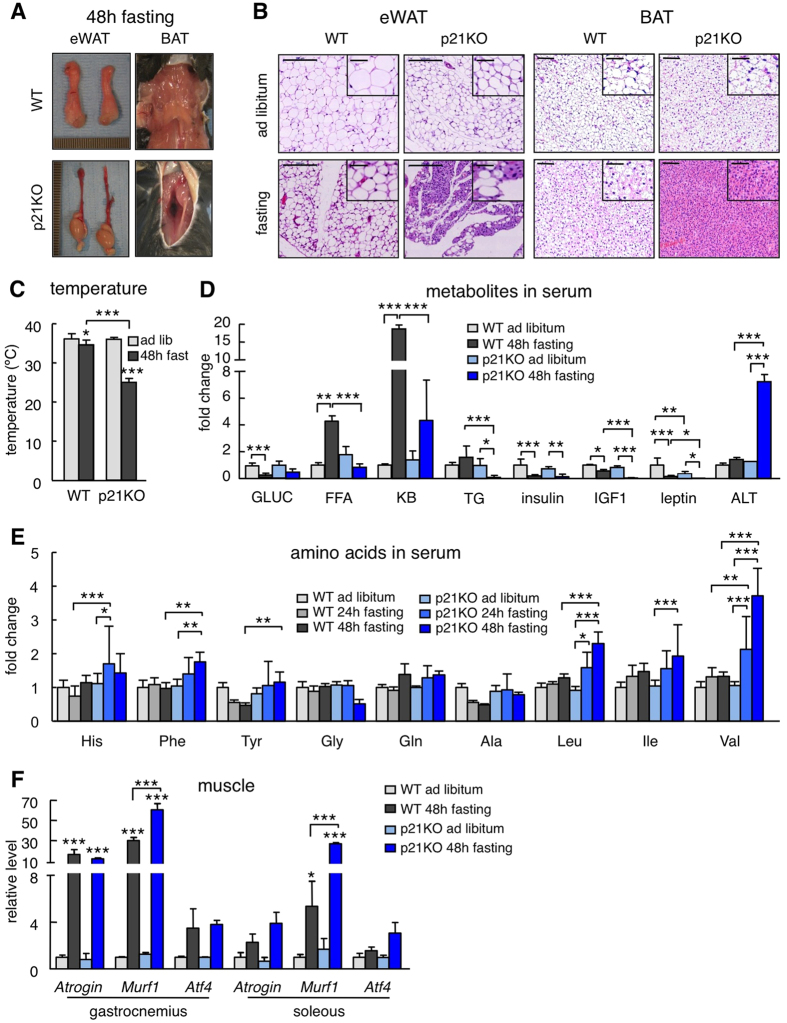
Impaired adaptation to prolonged fasting in p21^Cip1^ deficient mice. (**A**) Representative pictures of epididymal white adipose tissue (eWAT) and interscapular brown adipose tissue (BAT) of wild type and p21KO mice after 48 h fasting. (**B**) Representative pictures of H&E-stained sections of eWAT and BAT of ad libitum-fed or 48 h fasted WT and p21KO mice. Bars correspond to 0.2 mm. Bars in high magnification insets correspond to 50 μm. (**C**) Temperature of fed and 48 h fasted WT and p21KO mice. Temperature was measured with a rectal thermometer (n = 4–6 males, 12 weeks old). (**D**) Glucose (GLUC.), free fatty acids (FFA), ketone bodies (KB), tryglicerides (TG), insulin, IGF1, leptin and alanine aminotransferase (ALT) serum levels in WT and p21KO mice after 48 hours fasting or ad libitum feeding. (**E**) Relative level of the indicated amino acids in the serum of WT and p21KO mice after ad libitum feeding, 24 h or 48 h fasting. Measurements were performed by NMR (n = 4–5 males, 12 weeks old). (**F**) Relative expression of the indicated proteolytic factors in muscle of WT and p21KO mice after ad libitum feeding or 48 h fasting (n = 3 males, 12 weeks old). Values correspond to mean ± s.d. Statistical significance was determined by two-way ANOVA and Bonferroni post-hoc test: *p < 0.05, **p < 0.01, ***p < 0.001. See also Figure S1.

To test the role of p21 in metabolic adaptation to fasting, *p21*-null (p21KO) mice were fasted for 48 h. We did not observe obvious differences after 24 h of fasting, which is consistent with a previous report^5^. Remarkably, at 48 h, p21KO mice presented important differences compared to their controls (WT). In particular, p21KO mice were extremely stressed or lethargic, although their weight loss was comparable to that of WT mice (Figure S1A). Upon necropsy, all WT mice still retained white adipose tissue (WAT) and interscapular brown adipose tissue (BAT) (Fig. 2A). In contrast, p21KO mice had remnants of WAT and BAT (Fig. 2A). Histologically, the size of p21KO adipocytes was dramatically reduced compared to controls (Fig. 2B). Mice deficient in p21 presented a remarkable drop in body temperature (rectal) upon 48 h of fasting (Fig. 2C).

A number of metabolic adaptations were significantly altered in fasted p21KO mice. In particular, serum free fatty acids (FFA), triglycerides (TG) and ketone bodies (KB) were much lower than in WT mice (Fig. 2D; Figure S1B; Table S1), which suggest that p21KO mice exhaust lipid stores prematurely compared to WT mice. The levels of IGF1, insulin and leptin were also significantly decreased in p21KO mice (Fig. 2D; Table S1). The above alterations were accompanied by hepatic damage, as reflected by the serum levels of alanine aminotransferase (ALT) (Fig. 2D). Regarding the serum levels of amino acids, fasted p21KO mice presented higher levels of histidine, phenylalanine, tyrosine, leucine and valine (Fig. 2E). Interestingly, we also observed higher levels of expression of *Murf1* in skeletal muscle, which encodes a key ubiquitin ligase for muscle protein degradation during fasting (Fig. 2F). We interpret that the enhanced muscle proteolytic activity of p21KO mice compensates their premature loss of lipid stores.

**Figure 3:**
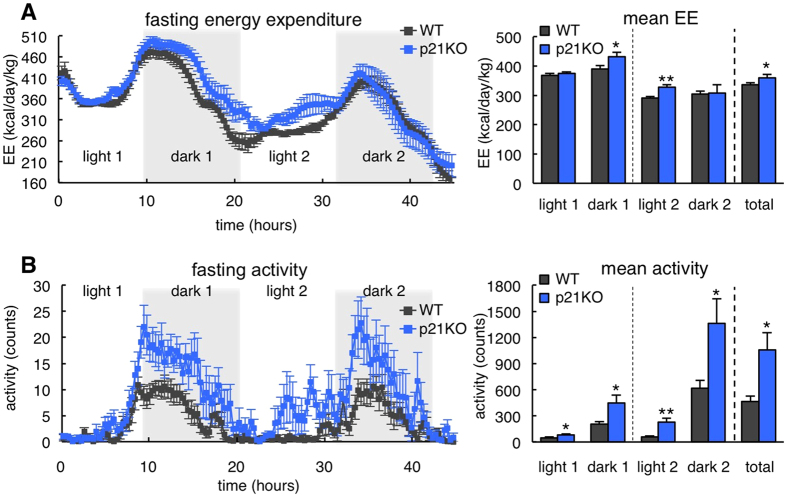
Increased energy expenditure and activity in p21^Cip1^-deficient mice during fasting. (**A**) Left, energy expenditure (EE) of WT and p21KO mice during 48 h of fasting. Right, mean EE of WT and p21KO mice at the indicated periods (n = 8 male mice, 12 weeks old). (**B**) Left, activity of WT and p21KO mice during 48 hours of fasting. Right, mean activity of WT and p21KO at the indicated periods under fasting conditions (same mice as in panel A). Values correspond to average ± s.e.m. Statistical significance was determined by the two-tailed Student’s t test: *p < 0.05, **p < 0.01. See also Figure S2.

The above observations suggest that p21KO mice exhaust their energetic reserves prematurely during fasting. To directly evaluate this, we measured energy expenditure (EE) in WT and p21KO mice during 48 h of fasting (Fig. 3A). Notably, p21KO mice presented higher levels of EE during the total period of fasting, and particularly during the first dark and second light periods (Fig. 3A). No significant differences were observed in EE under normal feeding conditions (Figure S2A). Fasting induces behavioral changes that reflect in elevated locomotor activity^12^. Interestingly, p21KO mice showed a significant increase in activity during the entire period of fasting (Fig. 3B), while no differences were observed under feeding conditions (Figure S2B). Together, we conclude that in the absence of p21, mice do not adapt efficiently to energy deprivation and exhaust prematurely their nutrient stores.

### Global transcription changes in p21^Cip1^ deficient mice

**Figure 4:**
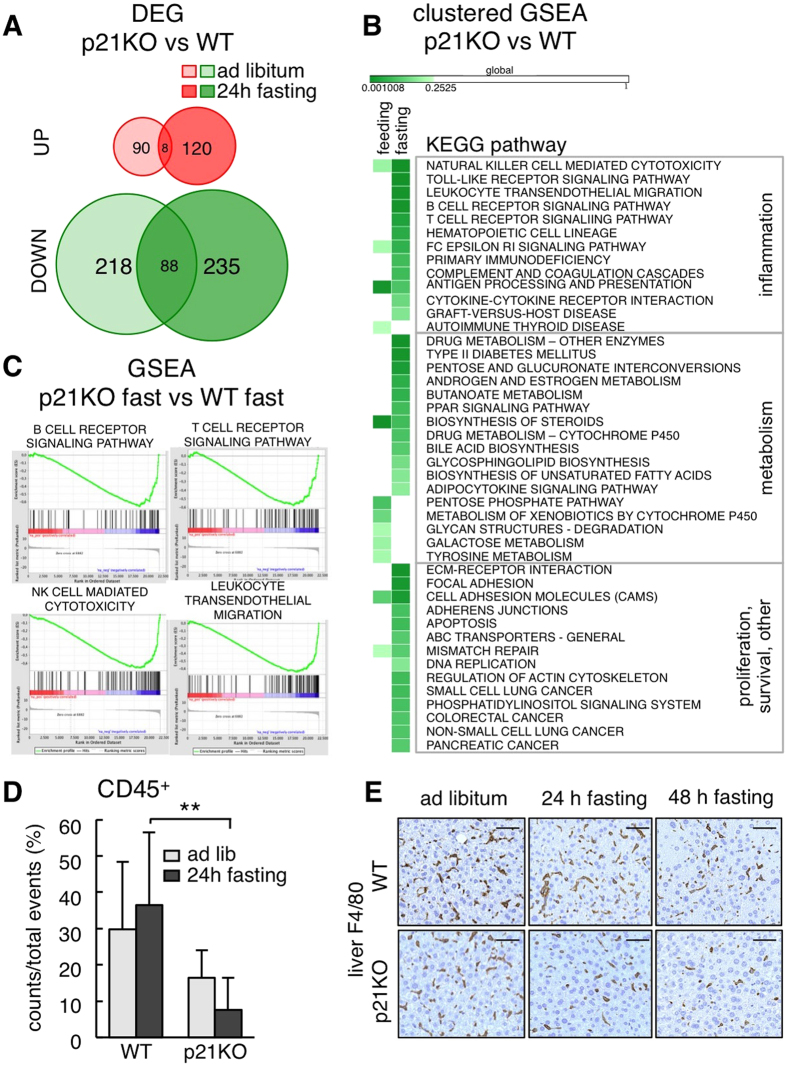
Global transcription changes in p21^Cip1^-deficient mice. (**A**) Differentially expressed genes (DEG) in the liver of p21KO compared to WT mice under ad libitum feeding conditions and upon 24 h fasting. Data were obtained from RNA-Seq analysis of 2–3 male mice per group. Red circles correspond to upregulated and green circles to downregulated genes. Values were considered significant when FDR value was q < 0.05. (**B**) Heat map of significantly altered pathways in the liver in the same samples as in panel A. Results were obtained from GSEA and considered significant when FDR < 0.25. (**C**) Representative GSEA plots of immune-related pathways downregulated in fasted p21KO mice compared to fasted controls. FDR < 0.25. (**D**) Relative amount of CD45^+^ immune cells in the liver of ad libitum fed and 24 h fasted WT and p21KO mice. Values correspond to average ± s.d. (n = 6 males, 12–16 weeks old). (**E**) Representative pictures of F4/80 stained liver sections of WT and p21KO mice under feeding conditions, 24 h and 48 h fasting. Bars correspond to 50 μm. Statistical significance was determined by two-way ANOVA and Bonferroni post-hoc test: **p < 0.01. See also Figure S3.

To gain insight into the mechanisms responsible for the defective adaptation of p21KO mice to prolonged fasting, we obtained the liver RNAseq profiles of WT and p21KO mice under standard feeding conditions and after 24 h of fasting (n = 2–3 per group). We chose 24 h of fasting to capture early defects in p21KO mice, prior to the severe phenotypes observed at 48 h. A total of 451 genes (128 UP and 323 DOWN) were differentially expressed (FDR *q* < 0.05) between p21KO and WT livers after 24 h of fasting and 96 of these genes (8 UP and 88 DOWN) were already differentially expressed under *ad libitum* feeding (Fig. 4A; Table S2). Gene-set enrichment analysis (GSEA) of pathways (KEGG) indicated an abundance of downregulated pathways related to inflammation in fasted p21KO livers (Fig. 4B,C; Table S3). One of the major consequences of fasting and dietary restriction is a general decrease in the number of infiltrating leukocytes and markers of inflammation^13^,^14^,^15^. To directly measure this, we quantified leukocyte (CD45^+^ cells) infiltration in the liver and we observed a significant reduction in fasted p21KO livers compared to fasted WT controls (Fig. 4D). This was further substantiated by measuring the mRNA levels of genes related to the immune system (Figure S3A), and by staining liver infiltrating macrophages with F4/80 (Fig. 4E). Of note, the relative proportion of leukocyte sub-populations was not altered in p21KO livers relative to WT controls (Figure S3B). We interpret that the reduced inflammation observed in p21KO mice further reflects the aggravated consequences of fasting in these mice.

### Decreased PPARα activity in the liver of p21^Cip1^ deficient mice

**Figure 5:**
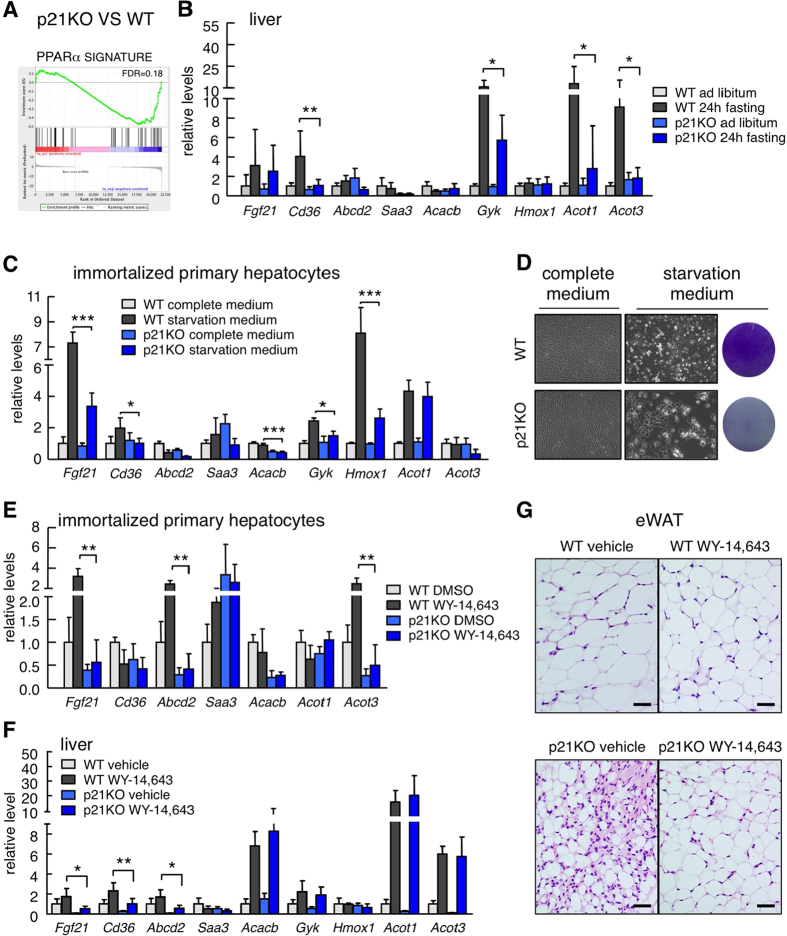
Decreased PPARα activity in the liver of p21^Cip1^-deficient mice. (**A**) GSEA plot of the PPARα signature in the liver of 24 h fasted p21KO mice compared to control mice. FDR = 0.18. (**B**) Relative mRNA levels of PPARα target genes in the liver of WT and p21KO mice under ad libitum feeding conditions and upon 24 h fasting (n = 6 per group, 12 weeks old male mice). (**C**) Relative mRNA levels of PPARα target genes expressed in WT and p21KO immortalized primary hepatocytes after culture in complete (DMEM + 10% FBS) or starvation medium (glucose-free and serum-free DMEM) for 24 h. The experiment was performed in 5–6 biological replicates (n = 5–6). (**D**) Left panel, representative pictures of WT and p21KO large-T immortalized primary hepatocytes cultured for 48 h in complete medium (DMEM + 10% FBS) or starvation medium (glucose-free and serum-free DMEM). Right, representative pictures of WT and p21KO plates stained with crystal violet after 48 hours culturing in starvation medium. The experiment was repeated three times and each time in three biological replicates with similar results. (**E**) Relative expression of PPARα target genes in WT and p21KO immortalized primary hepatocytes cultured in complete medium and treated with DMSO or with 10 μM of the PPARα agonist WY-14,643 for 24 h. The experiment was performed twice and each time in three biological replicates with similar results. One representative experiment is shown (n = 3). (**F**) Relative expression of PPARα target genes in the liver of WT and p21KO mice 6 h after treatment with vehicle or 75 mg/kg WY-14,643 by gavage. Mice were previously fasted overnight and maintained without food during the experiment. (**G**) Representative fields of epididymal white adipose tissue of WT and p21KO mice treated with vehicle or 75 mg/kg WY-14,643 by gavage (4 treatments, see Methods) during a period of 48 h fasting (n = 3–4 per group, 14 weeks old male mice). Bars correspond to 30 μm. Levels of mRNA were normalized to *β-actin.* Values correspond to average ± s.d. Statistical significance was determined by two-way ANOVA and Bonferroni post-hoc test: *p < 0.05, **p < 0.01, ***p < 0.001. Only significant differences between WT and p21KO samples under the corresponding experimental conditions (fasting, WY-14,643) are shown. See also Figure S4.

We took note of the fact that “PPAR signaling” was one of the gene sets downregulated in fasted p21KO mice compared to WT mice (Fig. 4B). PPARα is a critical hepatic transcription factor activated in response to nutrient deprivation and required for fasting adaptation^16^,^17^. Indeed, GSEA using a PPARα signature^18^ further suggested a reduction in PPARα activity (Fig. 5A). To validate the RNAseq data, we measured directly the mRNA levels of well-established PPARα transcriptional targets. Interestingly, some PPARα targets were significantly downregulated in fasted p21KO livers compared to WT controls (Fig. 5B). A similar situation was observed in immortalized primary hepatocytes upon nutrient starvation (Fig. 5C). Moreover, after 48 h of serum and glucose starvation, cultures of immortalized p21KO primary hepatocytes underwent massive cell death, whereas WT control hepatocytes remained largely viable (Fig. 5D).

To directly address the impact of p21 on the functionality of PPARα, we used the PPARα agonist WY-14,643. Interestingly, the induction of some WY-14,643-responsive genes was impaired in the absence of p21, both in primary hepatocytes (Fig. 5E) and in overnight fasted mice (Fig. 5F). We wondered if the observed partial activation of PPARα by WY-14,643 in p21KO mice would be sufficient to normalize their fasting response. Remarkably, p21KO mice treated with WY-14,643 were protected from severe fat depletion (Fig. 5G) and serum FFA depletion (Figure S4) after 48 h fasting. We conclude that p21 contributes to the metabolic adaptation to fasting acting in part as a positive regulator of PPARα.

## Discussion

Here, we have addressed a general question regarding the involvement of tumor suppressors in the response to fasting. On one hand, fasting is a physiological stress that triggers complex metabolic adaptions. On the other hand, some of the most important tumor suppressors participate in stress signaling pathways that are activated to prevent proliferation under stressful conditions. To explore the possible role of tumor suppressors in fasting, we began by asking if their mRNA levels respond to fasting. Interestingly, *p21*, but not other tested tumor suppressors tested, was highly upregulated in multiple tissues upon fasting and independently of p53.

Of note, *p21*-null (p21KO) mice manifested profound defects in the adaptation to fasting. This was particularly evident after 48 h of fasting, when p21KO mice showed clear signs of energy exhaustion, including lower serum levels of triglycerides, free fatty acids, and ketone bodies, together with a dramatic reduction of IGF1 and leptin levels, and a significant reduction in body temperature. In addition, fasted p21KO mice presented elevated levels of amino acids in the serum and a significant upregulation of the ubiquitin ligase MURF1 implicated in the degradation of muscle proteins. Finally, we observed that p21KO mice had higher energy expenditure during the first 36 h of fasting. All these alterations are consistent with a premature exhaustion of nutrient stores.

Unbiased examination of the transcriptome of p21KO livers upon 24 h fasting, when the severe defects of fasting have not been manifested yet, further supported the idea that the absence of p21 results in an accelerated onset of the effects of fasting. In particular, fasting reduces the levels of tissue infiltrating leukocytes present under normal feeding conditions^13^,^14^,^15^, but this reduction was more profound in the case of p21KO livers. This analysis also pointed out a defective activation of some PPARα transcriptional targets in fasted p21KO livers. This was particularly interesting because PPARα is critical for fasting adaptation^16^,^17^. In fact, PPARα-deficient mice present an abnormal response to fasting with some similarities to p21KO mice, such as lower body temperature and lower serum ketone bodies^16^,^17^. The defective activation of a subset of PAPRα transcriptional targets was confirmed in fasted p21KO livers, as well as, in p21KO hepatocytes upon nutrient starvation or treatment with a PPARα agonist (WY-14,643). To test the role of defective PPARα in the misadaptation of p21KO mice to fasting, treatment of these mice with the PPARα agonist prevented their premature depletion of fat stores. We conclude that the tumor suppressor p21 contributes to fasting adaptation by acting as a positive regulator of PPARα.

## Methods

### Ethics statement

All animal procedures were approved by the CNIO-ISCIII Ethics Committee for Research and Animal Welfare (CEIyBA) and the Community of Madrid, and conducted in accordance to the recommendations of the Federation of European Laboratory Animal Science Associations (FELASA) and the institutional guidelines.

### Mouse experimentation

Mice were housed under specific pathogen free (SPF) conditions, at 22 °C, and with 12 hours dark/light cycles (light cycle from 8 am to 8 pm). Mice were fed with standard chow diet (Harlan Teklad 2018, 18% of fat-based caloric content) or fasted for 48 or 24 hours, with ad libitum access to water, when specified. Complete necropsies were performed in parallel when ad libitum and fasted mice were compared. All the mice used in this work are C57BL6J/Ola.Hsd males of 10–16 weeks of age. Temperature was measured using a rectal thermometer (Braintree Scientific). For administration of WY-14,643, mice were fasted overnight, orally treated by gavage with 75 mg/kg WY-14,643 (Sigma) dissolved in PEG300 (Sigma) and 10% N-methyl-2-pyrrolidone (Sigma) and sacrificed 6 hours later. When combined with 48 hours fasting, mice were treated four times with 75 mg/kg WY-14,643 by oral gavage (at the beginning of fasting and after 8, 24 and 32 hours of fasting).

### Cells

Primary hepatocytes were obtained from the liver of 3–5 day-old neonates as previously described^19^ and retrovirally immortalized with Large-T antigen (in vector pBABE-puro). Cells were maintained in complete medium consisting of DMEM supplemented with 10% fetal bovine serum (all by Gibco) and incubated in 20% O_2_ and 5% CO_2_ at 37 °C. When indicated, cells were nutrient starved in starvation medium consisting of glucose-free DMEM (Gibco) without FBS supplementation for 24 or 48 hours and/or treated with 10 μM forskolin (Sigma) for 5 hours, 10 μM CNIO PI3K inhibitor (PI3Ki)^20^ for 5 hours or the PPARα agonist WY-14643 (Sigma) for 24 hours. For crystal violet staining, cells were fixed in 1% glutaraldehyde for 10 minutes and stained with 0.1% crystal violet for 30 minutes.

### Histology and immunohistochemistry

Tissues were fixed overnight in formalin, embedded in paraffin blocks and sectioned. Tissue sections were stained with hematoxylin/eosin (H&E) or with anti-F4/80 (ABD Serotec, #MCA497) following standard procedures.

### Indirect calorimetry and activity

Indirect calorimetry was performed following standard methods using Oxylet System metabolic chambers (Panlab Harvard Apparatus). Mice (12–13 weeks old males) were acclimatized to the measurement cages for three days prior to data recording. Volume of consumed O_2_ (VO_2_) and eliminated CO_2_ (VCO_2_) was recorded in *ad libitum* fed mice for 48 hours. After that, food was withdrawn and data were recorded for the next 48 hours under fasting conditions with ad libitum water access. Room temperature was constantly kept at 21 °C and light/dark cycles were of 12 hours. Respiratory Quotient (RQ) was calculated as RQ = VCO_2_/VO_2_ from volumes of consumed O_2_ (VO_2_) and eliminated CO_2_ (VCO_2_) recorded every 24 minutes (8 simultaneous metabolic chambers). Energy Expenditure (EE) was calculated as EE = (3.815 + (1.232 × RQ)) × VO_2_ × 1.44). Moreover, we recorded mouse activity in time intervals of 20 minutes during the whole measurement period.

### Serum analyses

For serum glucose determination, blood was collected from the tail tip and glucose was measured using Glucocard strips (A. Meranini Diagnosis). For serum isolation, blood was collected from post-mortem heart puncture. Serum insulin (Ultra Sensitive Mouse Insulin ELISA kit; Crystal Chem Inc.), Igf1 (Mouse/Rat IGF-1 ELISA; Demeditec), leptin (Crystal Chem Inc.) and adiponectin levels (Invitrogen) were measured by ELISA following the manufacturer’s instructions. Serum triglycerides (Serum Triglyceride Determination Kit; Sigma), free fatty acids (Wako NEFA C Kit; Wako Chemicals) and ketone bodies (Autokit 3-HB; Wako Chemicals) were quantified by colorimetric assay. Alanine aminotransferase (ALT) levels were measured using the ABX PENTRA 400 clinical chemical analyser (Horiba ABX Diagnostics).

### RNA analyses

Total RNA from tissues or cells was extracted using TRIZOL (Invitrogen). cDNA synthesis was performed with 1–2 µg of RNA using iScript First Strand cDNA synthesis kit (BioRad #170-8891). Quantitative real time-PCR (qRT-PCR) was carried out using *GoTaq Q-PCR* Master Mix (Promega) in a 7500 Fast Real-Time PCR System (Applied Biosystems). Reactions were performed in triplicate and normalized by *β-actin* or *Gapdh* expression in the case of the muscle. Primer sequences are described in Supplementary Information (Table S4).

### RNA-seq-based transcriptional profiling

Livers were snap-frozen in liquid nitrogen and RNA was prepared using Trizol (Invitrogen) and RNeasy Mini kit (Qiagen) following the manufacturer’s instructions. RNA Integrity Number (RIN) was in the range of 9.1–9.5 (Agilent 2100 Bionalyzer). 2–8 ng of total RNA was used to synthesize the cDNA (SMARTer Ultra Low Input RNA Kit, version 3, Clontech #634848). After amplification with SeqAmp DNA Polymerase (Clontech), 10 ng of cDNA was used to prepare the adaptor-ligated library following the “TruSeq DNA sample preparation guide” (part #15005180). The resulting cDNA libraries were sequenced for 50 bases in a single-read format (Illumina HiSeq2000). Reads were aligned to the mouse genome (GRCm38/mm10) with TopHat-2.0.10^21^ using Bowtie 1.0.0^22^ and Samtools 0.1.19^23^, allowing two mismatches and five multihits. Transcripts assembly, estimation of their abundances and differential expression were calculated with Cufflinks 2.2.1^21^, using the mouse genome annotation data set RCm38/mm10 from the UCSC Genome Browser. Gene Set Enrichment Analysis (GSEA) was performed using annotations from the KEGG, Reactome and NCI databases. Genes were ranked using the t statistic. After Kolmogorov-Smirnoff correction for multiple testing, only those pathways bearing a FDR < 0.25 were considered significant. Enrichment plots were also obtained with GSEA and ranked according to their enrichment score (ES).

### Flow cytometry

Cells from ad libitum fed or 24 hour fasted WT and p21KO mice (12–16 weeks old, n = 6) were isolated from spleen and liver by disaggregating tissues through a 70 μm or 100 μm strainer respectively. Following erythrocyte lysis by Red Blood Cell Lysis Buffer (Qiagen), immune cells from the liver were further isolated using a 38–70% Percoll gradient (GE Healthcare). Cells were then blocked in Fc block (CD16/CD32, BD Biosciences #553141) diluted 1:400 on ice for 30 minutes and incubated with the following conjugated antibodies for 1 hour at 4 °C in a rotating platform: CD8a-FITC (eBioscience #11-0081-82), CD4-PE (eBioscience #12-0041-82), CD45-PerCP (Biolegend #103130), CD11b-PerCP/Cy5.5 (eBioscience #45-0112-82), Gr1-PECy7 (eBioscience #25-5931-82), NK1.1-PECy5 (Biolegend #108715), F4/80-AF647 (eBioscience #12-0041-82), CD3-AF700 (eBioscience #56-0032-82), CD19-APC/EF780 (eBioscience #47-0193-80) and B220-APC/EF780 (eBioscience #47-0452-82). Splenocytes were used as Fluorescence Minus One (FMO) to gate cell populations and commercial anti-mouse or anti-rat IgG beads (BD Biosciences #552843 or #552844) to compensate for fluorochrome spectral overlap. We used pulse processing to exclude cell aggregates and an amine reactive live/dead dye (Aqua, Invitrogen) to exclude dead cells. At least 10,000 cells from the CD45 gate were collected. Cells were analyzed in an LSR-Fortessa (BD Biosciences; FACS Diva software) and all data analyzed using FlowJo v9.6.2 software (Trestar, Oregon).

### Nuclear magnetic resonance

Mice blood for serum isolation was collected by post-mortem heart puncture. After coagulation in ice, 100 μl of serum were mixed with 100 μl ice-cold 2X PBS buffer in deuterium oxide (D_2_O), centrifuged for 10 min at 16000 × g and transferred to a 3 mm NMR sample tube. NMR spectra were recorded at 20 °C in 9 mins on a Bruker Avance 700 MHz spectrometer. Metabolite levels in serum samples were determined from the integrals of the most resolved and largest signals of each metabolite (for example methyl groups of lactate, alanine and pyruvate, and H4 proton of glucose) in 1D ^1^H NMR spectra acquired with a transversal relaxation filter (CPMG of 200 ms, τ = 0.4 ms) that attenuates the fast relaxing signals of macromolecules (proteins, lipids and lipoproteins) signals and optimises the NMR signals of low mass metabolites for their quantification^24^. Thus, the obtained values of integrals are not absolute concentrations but relative concentrations in arbitrary units (AU).

### Statistical analyses

Values are expressed as mean ± s.d. (the only exception is the calorimetry and activity data, Figs 3 and S2, where values correspond to the mean ± s.e.m.). Comparisons between two groups or conditions (Figs 1A–D and 3) were performed using unpaired two-tailed Student’s t-test. All the other analyses correspond to multiple comparisons and were performed using two-way ANOVA and Bonferroni post-hoc test. Differences with *P* values of <0.05 were considered to be statistically significant (*p < 0.05, **p < 0.01, ***p < 0.001). Statistical analyses were performed using Excel or GraphPad Prism software.

## Additional Information

**How to cite this article**: Lopez-Guadamillas, E. *et al.* p21^Cip1^ plays a critical role in the physiological adaptation to fasting through activation of PPARα. *Sci. Rep.* **6**, 34542; doi: 10.1038/srep34542 (2016).

## Supplementary Material

Supplementary Information
