# Supplementary material for: p21Cip1 plays a critical role in the physiological adaptation to fasting through activation of PPARα
Source: Sci Rep. 2016 Oct 10;6:34542. doi: 10.1038/srep34542 (PMC5056372; doi:10.1038/srep34542)
Supplement: Supplementary Information [file srep34542-s1.pdf]

$p21^{Cip1}$ plays a critical role in the physiological adaptation to fasting through activation of PPARα

Elena Lopez-Guadamillas, Pablo J. Fernandez-Marcos,
Cristina Pantoja, Maribel Muñoz-Martin, Dolores Martínez, Gonzalo Gómez-López,
Ramón Campos-Olivas, Angela M. Valverde and Manuel Serrano

SUPPLEMENTARY INFORMATION

<u>**INVENTORY**</u>

SUPPLEMENTARY FIGURES AND LEGENDS (Figure S1 – Figure S4)

SUPPLEMENTARY TABLES (Table S1 – Table S4)

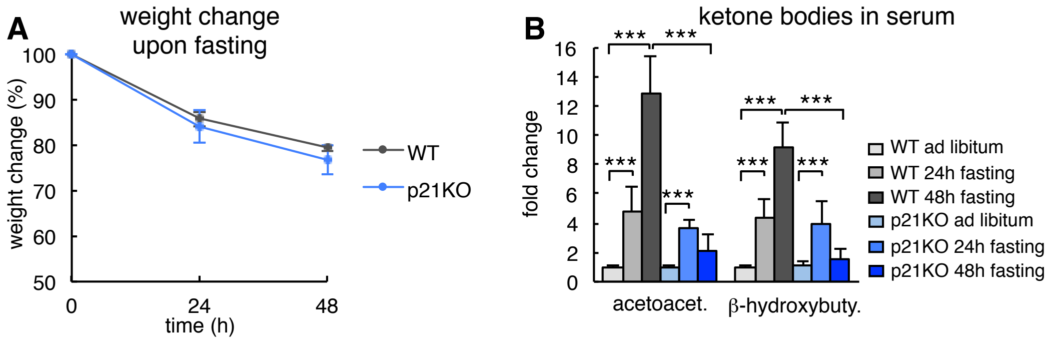

Figure S1 (related to Figure 2)

(A) Weight change of WT and p21KO mice after 24 and 48 h fasting (n=4 males, 12 weeks old).

(B) Relative serum ketones bodies (acetoacetate and β-hydroxybutyrate) in ad libitum fed, 24 h or 48 h fasted WT and p21KO mice. Measurements were performed by NMR (n=4-5 males, 12 weeks old).

Values correspond to average ± s.d. Statistical significance was determined by two-way ANOVA and Bonferroni post-hoc test: *** $p<0.001$.

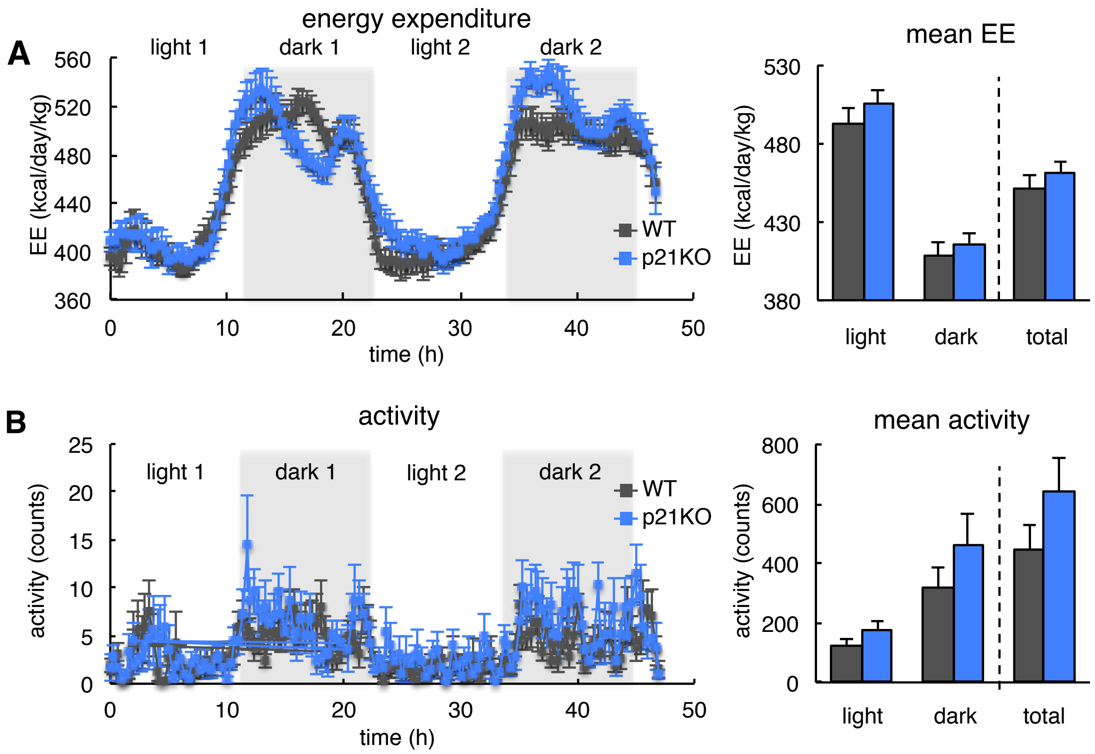

Figure S2 (related to Figure 3)

(A) Left, energy expenditure (EE) of WT and p21KO mice during 48 h under standard feeding conditions. Right, mean EE of WT and p21KO mice at the indicated periods (n=8 male mice, 12 weeks old).

(B) Left, activity of WT and p21KO mice during 48 hours under standard feeding conditions. Right, mean activity of WT and p21KO at the indicated periods.

Values correspond to average ± s.e.m.

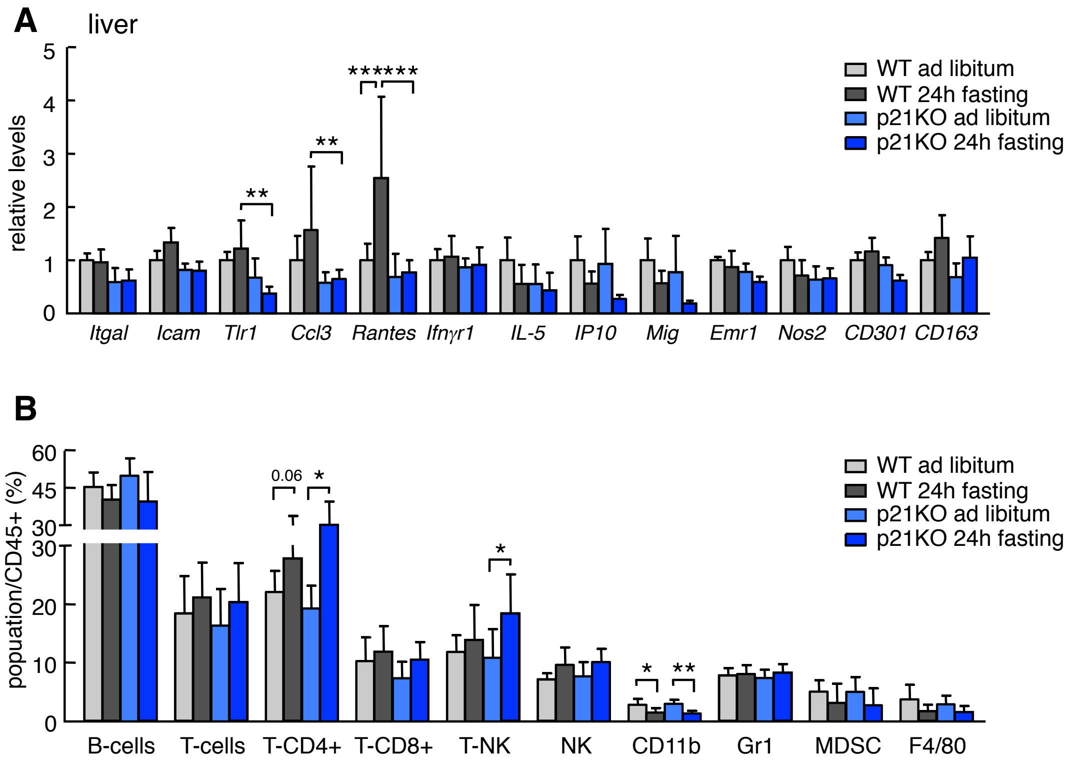

Figure S3 (related to Figure 4)

(A) Relative expression of the indicated genes related to inflammation in the liver of WT and p21KO mice under ad libitum feeding conditions or 24 h fasting (n=6, males, 12 weeks old). mRNA levels were normalized to *β-actin*.

(B) Percentage of the indicated immune cell populations relative to CD45+ leukocytes, in the liver of ad libitum fed or 24 h fasted WT and p21KO mice (n=6 males, 12-16 weeks old).

Values correspond to average ± s.d. Statistical significance was determined by two-way ANOVA and Bonferroni post-hoc test: *p < 0.05, **p<0.01, *** p<0.001.

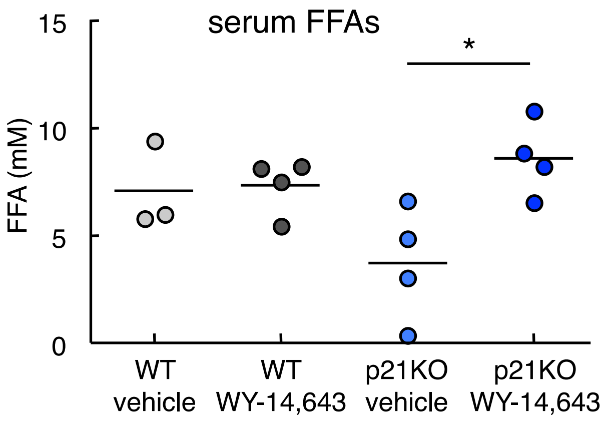

Figure S4 (related to Figure 5)

Serum free fatty acids in WT and p21KO mice treated with vehicle or 75 mg/kg WY-14,643 by gavage (4 treatments, see Methods) during a period of 48 h fasting (n=3-4 per group, 14 weeks old male mice). Values correspond to average ± s.d. Statistical significance was determined by two-way ANOVA and Bonferroni post-hoc test: *$p < 0.05$.

SUPPLEMENTARY TABLES

Table S1: metabolites in serum

	WT		p21KO	
	ad libitum	48h fasting	ad libitum	48h fasting
temperature (ºC)	36.15 ± 1.3	34.62 ± 1.2 *	36.0 ± 0.5	25.0 ± 1.0 *** ###
glucose (mg/dl)	122.75 ±14.27	43.0 ± 11.17 ***	135.8 ± 32.73	57.5 ± 11.38
free fatty acids (nM)	0.28 ± 0.05	1.20 ± 0.11 **	0.50 ± 0.17	0.23 ± 0.70 ###
ketone bodies (µM /l)	94.0 ± 8.9	1766.3 ± 110.3 ***	130.6 ± 63.0	410.5 ± 283.7 ###
triglycerides (mg/ml)	0.35 ± 0.07	0.55 ± 0.30	0.34 ± 0.17	0.03 ± 0.05 * ###
insulin (ng/ml)	0.78 ± 0.36	0.15 ± 0.07 ***	0.57 ± 0.12	0.10 ± 0.15 **
IGF1 (ng/ml)	901.25 ± 47.25	504.06 ± 97.24 *	741.5 ± 103.30	26.25 ± 32.10 *** ###
adiponectin (ng/ml)	83.39 ± 8.62	68.17 ± 11.25 **	94.22 ± 12.73	69.79 ± 21.31 *
leptin (ng/ml)	4.46 ± 2.37	0.65 ± 0.34 ***	1.53 ± 0.79 ##	0.13 ± 0.04 *** ##
ALT (u/l)	26.55 ± 4.07	37.77 ± 3.99	33.85 ± 0.21	192.97 ± 14.26 *** ###

* Significant difference relative to ad libitum control (two-way ANOVA and Bonferroni post-hoc test): *p < 0.05, **p<0.01, ***p<0.001).
\# Significant difference relative to WT control (two-way ANOVA and Bonferroni post-hoc test): #p < 0.05, ##p<0.01, ###p<0.001).

Table S2: RNA-seq DEG data (q < 0.05)

UPREGULATED GENES IN p21KO

**p21KO ad lib *vs.* WT ad lib**	**p21KO *vs.* WT (both)**	**p21KO fast *vs.* WT fast**
Arrdc3	8430408G22Rik	Lepr
Egr1	Glo1	Cyp2b10
Mup17	Igfbp2	Pdk4
Zbtb16	Rgs16	Acmsd
Dynlt1c,Dynlt1f	Pla2g12a	Cbs
Efna1	Agxt2l1	Hamp2
Dynlt1b	Cobll1	Adcy1
Junb	Wnk4	Ppp1r3g
Dynlt1a		Cyp17a1
Spry4		St5
Nr0b2		Hamp
Eno1,Gm5506		Hspb1
Bcl6		Mt1
Pim1		Mt2
Syvn1		3930402G23Rik
Hsd17b6		Cend1
Il6ra		Ddit4
Nfxl1		Rab44
Mup5		Hba-a2
Rnft2		Txnip
Il1r1		Arl4a
Rnf39		Atf5
Zkscan1		Reps1
Dnajb9		B930025P03Rik
Acsm3		Sulf2
Nr1d1		Krt23
Mup6		Serpine2
Zfp36		Igfbp1
Tgif1		Cpt1b
Pcbp2		Sdsl
Tmem39a		Dnajb2
Selenbp2		Il1rn
Osgin1		Kif21a
Pim3		Sik1
Zfp862		Fam134b
Gdf15		Fgf21
Aadat		Rnf167
Clec2h		Plin5
Mtss1		Chkb
Fzd8		Ablim3
Enpp2		Camk2b
Slc13a2		Scara5
Atp6v0c		1700017B05Rik
Sfpq		4930452B06Rik
Arhgef26		E030018B13Rik
Nox4		Tacc2
Creb3l2		Slc41a3

Gigyf2
Golph3l
Ppp2r5e
Pabpc4
4933426M11Rik
Gfpt1
Irs2
G0s2
Xlr3a
Fam193b
Arhgap32
Mup9
Hbb-b1,Hbb-b2
Lifr
Herpud1
Trib3
Cela1
Cdk13
Ces2c
Slc25a34
Map3k5
Eif2ak3
Ep400
Synj2
Cyp2u1
Anks1
Xlr3b
Ehmt1
Nfix
Dsg1c
Ppargc1b
Whsc1l1
Ass1
Hyou1
Fzd5
Myo1e
Sun2
Trib1
Cys1
Rhobtb1
Ppp1r3c
Fam107b
Cpeb2

Arntl
Accn5
Trp53inp1
Cyp2b13
Josd2
Maff
Hilpda
Dusp8
Ppargc1a
Nrg4
Map3k6
Parp16
Lpin2
Rabggtb
Slc16a10
Rab30
Arhgef40
Arl15
Sorbs3
Cdc42ep5
Aldoa
Tubb2b
Srrm4
Fbxo6
Myom1
Derl3
Grtp1
Cyp39a1
Tbc1d8
Abtb2
Rbpms
Fbxo31
Peg3
Nnmt
Lman2l
Eif4ebp3
Clpx
Nedd4l
Slc20a1
Sf1
Rhbdd2
Tubb2a
Cgref1
Echdc2
Cep110
BC057022
Cdkn1a
Slc25a33
Acacb
Lepre1
Mcc
Pde4c
Peg3as

		Creld2
		Atoh8
		Zfhx2
		Tnfrsf12a
		Rps4y2
		Chpf
		Paip1
		Il3ra
		Tnk2
		Pogk
		Vopp1
		Atf3
		Kctd15
		Ccnf
		Gadd45b
		Eppk1
		Elmod3
		2010003K11Rik
		Nr4a1
		P4ha2

DOWNREGULATED GENES IN p21KO

**p21KO ad lib *vs.* WT ad lib**	**p21KO *vs.* WT (both)**	**p21KO fast *vs.* WT fast**
Fgl1	Apcs	Irf7
Gadd45g	Lcn2	Socs2
Mfsd2a	Orm2	Acot3
Mt1	Saa1	Stat1
Mt2	Saa2	Spp1
Myc	Saa3	H2-Aa
Ppp1r10	Tiam2	Itih4
Hcn3	Apol9a	Ndrg2
Cabyr	Trim30d	Ifi27l1
Apoa4	Pfkfb3	Entpd5
Mvd	Igtp	Sorbs2
Lrg1	Ly6e	H2-Eb1
Fbf1	Tymp	9030619P08Rik
Fdps	Tgtp1	Lyz2
Mid1ip1	Tff3	H2-Ab1
Orm3	Samd9l	Col3a1
1810011O10Rik	Cish	Apol7a
Slc3a1	Ifi44	Clec7a
Cyp4a14	Marco	Chpt1
Acacb	Gbp6	Cd5l
Pmvk	Tgtp2	Tbc1d24
Ppp1r3b	Ifit1	Ly6d
Acsl3	Ifi27l2b	Slc44a3
Nedd9	Gbp10	Cd24a
Apol9b	Thrsp	Arhgap19
Gm5506	Gm4070	Rbfox2
Serpina4-ps1	Axl	Emr1

Usp18
Ucp2
Prepl
Nnmt
Prg4
Tmem176b
Chrna4
Dnajb2
Tmem51
Pcsk9
H2-T9
Fads3
Hmgcs1
Steap4
Orm1
Tap1
Hmgcr
Atp11a
Hbb-b2
Aacs
2010003K11Rik
Extl1
Fam65b
Smpd3
Gm8801
Rgs3
Shisa5
Eif4e3
Oasl1
Sqle
Tgm1
Gpr110
Trafd1
Fgfr3
Pml
Lbp
Gm14403
Lss
Sco2
Cdhr5
Cyp26a1
Tmie
Fdft1
Unkl
Ifit3
Adar
Acnat2
Rasgef1b
Dhx58
Cd151
Srxn1
Tcirg1
Jak3
Cd36
Mpeg1
Pklr
Rtn4
Tmem176a
Prlr
Col15a1
Tifa
Wdfy1
Zbp1
Mmp19
Lrtm1
Mmd2
Ttc23
Psmb9
Gbp3
Erdr1
Wsb1
Gm4841
Aqp8
Trim30a
Psmb8
Ifi47
Itgal
Il18bp
Gbp7
Parp14
Hacl1
Slc13a5
Slc13a3
Fam198a
Oas1a
Gm12250
Sirpa
Fam46a
Gas6
Irgm2
Dmpk
Trim12c
St6gal1
C1qc
Gstt3
Pls1
AW112010
Csf1r
Ctss
C1qa
Irgm1
Tspan4
Cxcl9
Robo1
Pla2g7
Rtp4
Abcd2
Cybb
Lgals1
Acly
Cd97
Arhgef9
Iigp1
Vcam1
Samhd1
Tlr12
Paqr9
Ces2c
1110020G09Rik
Lyz1
Klhdc7a
Ubp1
Slc39a4
Zfp207
Arap1
Oxr1
Osbpl3
Uap1l1
Olfml1
Col1a1
Siglec1
Immt
Alas2
Slc41a2
Ptprc
Lima1
Lilrb4
Gbp2
Aoah
Flnb
Keg1
Cd52
Cxcl12
Nckap1l
Tmsb4x
Myof
Sdc3
Gigyf2
Ncald
Ccl5
Scd4
Cxcl13
Pik3r1
Ubd
Ptprg
Lrit1
Il2rg
AF251705
Scd2

Serpina7
Ccrn4l
S100a10
Gm7120
Isg15
Trim34a
Cebpe
Fasn
Gstm4
Hn1l
Aldh1b1
Acss2
1300015D01Rik
Gm16551
G6pc
Fam53b
Mtmr11
Slc16a5
Spsb3
Hspb1
Pnpla3
Ddc
Rxrg
C1qtnf1
Irf9
Pgd
Bud13
Gck
Tagap1
Mlkl
Tubb2a
Naip2
Pltp
Serinc2
Ildr2
Josd2
Tor1aip2
Ddx60
Insc
Rap1gap
Isyna1
Hsp90aa1
Slc37a1
Zc3hav1
Gtpbp2
Tceal8
Sc4mol
Tnfsf10
Tgfbr2
Clstn3
Csrp3
Gm19619
Pvrl2
Gsdmd
A230050P20Rik
Tnfrsf19
Kifc3
Gm8979
Cd74
Wwtr1
Fam84b
Ank3
Arhgap25
Rbm3
Ces1g
Scara3
Ghr
Vwa5a
Slc7a8
Cib3
Ces2d-ps
Cyp2a4
Rps6ka1
Nat8
E330011O21Rik
Rasa4
Klf13
Lyn
Ccr5
Scd3
Sorbs1
Ugt1a9
Clec4a3
Ociad2
Nr3c2
Adcy7
Irf8
Erbb2ip
Rnf152
Clec12a
Aif1
Tlr13
Stk10
Ces2e
Nnt
Lgals3
Cyp3a25
C1qb
Rcbtb2
Zfp809
Lpcat2
Cml5
AI182371
Gbp9
Myo1f
Abcg3
Nr1h4
Pld4
Lphn2
Fyb
Phldb2
Iqgap1
Fam55b
B3galt1

Dock9
Srp54b,Srp54c
Bst2
Znrf1
Gm7694
Mdp1
Fam47e
Pdzk1ip1
Mcm6
Gnat1
Gtf2ird1
St6galnac6
D14Ertd449e
Serpina11
Ctgf
Nlrc5
Sgsm1
Fam25c
Ube2h
Herc6
Mvk
Got1
Fam129b
Ifitm2
Zbtb4
Lgals3bp
Nrp2
Aig1
Dhcr7
I830012O16Rik
Steap2
Cyp4a10
Sun1
St5
Cpne8
Tmem98
Zfp259
Trim21
Plac8
Oasl2
Grn
Jub
Rras
Xaf1
Nsdhl
Slc25a25
Trim12a
Cyb561
1700024P16Rik
Agpat9
Filip1l
Mtap7d1
Sall2
Tstd1
Csf1
Cyp2c50
Dcun1d1
Cd68
Klra2
Ahnak
Gda
Gbp8
Lair1
Hpgds
Acbd5
Fgd2
Cenpl
Arhgap30
Acot4
Rasal2
Lpl
Coro1a
Gbp1
Igfals
Pnldc1
Eif4g3
Hck
Gbp11
Blnk
Capg
Cyp2a5
Hk3
Lgmn
Taok3
Rdh9
Cldn2
Slc15a3
Tfrc
Ly86
Hnmt
Gp49a
Cxcl16
Tyrobp
BC013712
Fam129a
Laptm5
Anxa2
Dut
C730036E19Rik
Syk
Unc119
Adhfe1
Cd48
Lilrb3
Slc2a9
Cd180

Cyp17a1
Oas1g
Sfxn5
Arhgef19
Gm14431
Ifitm3
Mfge8
Tap2
Ggt6
Elovl6
Gpc1
Cldn14
Golm1
Htatip2
Tm6sf2
AB124611
Ddhd1
Slc17a1
Fam73b
Hba-a1,Hba-a2
Srp54a
Cmpk2
Gm5480
D730039F16Rik
Alas1
Elovl5
Abtb2
Ppap2c
Ppl
Ang
Litaf

Lsp1
Cbr1
Rgn
Itgb2
Adam33
Phka2
Ptprd
Timd4
Gstm3
Gm8989
Adra1b
Fabp5
B430306N03Rik
Herc3
Efha1
Pira2
Gm4951
Idi1
Parp12
S100a11
Magi1
Mpp1
Trim24
Hmox1
Mapkapk3
Lrrc25
Tmtc4
Aifm3
Gng2
Evi2b
Mthfd1l
Mmp15
Clec4a1
1600014C10Rik
Clec4n
Folr2
Aatk
Abr
Marcks
Gna14
Hipk2
Mta3
Cxcl10
Scd1
Ctla2b
Nfib
Vsig4
Cd276
Cyp3a16

Table S3: RNA-seq GSEA data

downregulated in
p21KO ad libitum *vs.* wt ad libitum

KEGG gene set	FDR
biosynthesis of steroids	0.001
antigen processing and pesentation	0.010
proteosome	0.032
glycolysys/gluconeogenesis	0.062
pentose phosphate pathway	0.106
cell adhesion molecules	0.130
galactose metabolism	0.147
natural killer cell mediated cytotoxicity	0.203
tyrosine metabolism	0.204
adipocytokine signaling pathway	0.214
glycan structures-degradation	0.216
primary immunodeficiency	0.218
DNA replication	0.231
autoimmune thyroid disease	0.234

downregulated in
p21KO 24h fasting *vs.* wt 24h fasting

KEGG gene set	FDR
natural killer cell mediated cytotoxicity	0.002
ECM-receptor interaction	0.009
leucocyte transendothelial migration	0.009
drug metabolism-other enzymes	0.009
type II diabetes mellitus	0.012
B-cell receptor signaling pathway	0.012
toll-like receptor signaling pathway	0.012
focal adhesion	0.017
cell adhesion molecules	0.022
butanoate metabolism	0.028
T-cell receptor signaling pathway	0.039
fc epsilon ri signaling pathway	0.058
pentose and glucuronate interconversions	0.061
primary immunodeficiency	0.068
androgen and estrogen metabolism	0.070
apoptosis	0.088
porphyrin and chlorophyll metabolism	0.089
complement and coagulation cascades	0.090
regulation of actin cytoskeleton	0.092
biosynthesis of steroids	0.092
hematopoietic cell lineage	0.093
pancreatic cancer	0.093
PPAR signaling pathway	0.094
phosphatidylinositol signaling system	0.099
adherens junction	0.101
bile acid biosynthesis	0.104
antigen processing and presentation	0.108
colorectal cancer	0.114
small cell lung cancer	0.114
gamma hexachlorocyclohexane degradation	0.117
dentatorubropallidoluysian atrophy	0.119
mismatch repair	0.120
drug metabolism-cytochrome P450	0.121
drug metabolism-cytochrome P450	0.121
DNA replication	0.123
cytokine-cytokine receptor interaction	0.148
biosynthesis of unsaturated fatty acids	0.161
non-small cell lung cancer	0.171
graft-versus-host disease	0.176
glycosphingolipid biosynthesis	0.179
ABC transporters	0.180
metabolism of xenobiotics by cytochrome P450	0.189

Table S4: qRT-PCR primers

PCR primers for mouse transcripts

Primer	Forward sequence 5' → 3'	Reverse sequence 5' → 3'
β-Actin	GGCACCACACCTTCTACAATG	GTGGTGGTGAAGCTGTAGCC
Gapdh	TTCACCACCATGGAGAAGGC	CCCTTTTGGCTCCACCCT
Emr1	TGACTCACCTTGTGGTCCTAA	CTTCCCAGAATCCAGTCTTTCC
G6pc	ACTGTGGGCATCAATCTCCT	AGGTGACAGGGAACTGCTTT
Pgc1α	GGGTTATCTTGGTTGGCTTTATG	AAGTGTGGAACTCTCTGGAACTG
$p21^{Cip1}$	GTGGGTCTGACTCCAGCCC	CCTTCTCGTGAGACGCTTAC
$p16^{Ink4a}$	TACCCCGATTCAGGTGAT	TTGAGCAGAAGAGCTGCTACGT
$p19^{ARF}$	GCCGCACCGGAATCCT	TTGAGCAGAAGAGCTGCTACGT
$p27^{Kip1}$	TCAAACGTGAGAGTGTCTAACG	CCGGGCCGAAGAGATTTCTG
p53	GCGTAAACGCTTCGAGATGTT	TTTTTATGGCGGGAAGTAGACTG
Fgf21	GTGTCAAAGCCTCTAGGTTTCTT	GGTACACATTGTAACCGTCCTC
CD36	ATGGGCTGTGATCGGAACTG	TTTGCCACGTCATCTGGGTTT
Abcd2	TGTGGAGCAGCTGTGGACTA	ATCAGCTCCAGAGGCCAGTA
Saa3	TAAAGTCATCAGCGATGCCAGAG	CAACCCAGTAGTTGCTCCTCTTC
Acacb	GTATCCGCAAGGCTGAGAGT	GTTCTGGGCCAGCTTCATTA
Gyk	TGAAGAAAGCGAAATCCGTTACT	CCCAAAGGCAGACTACAGAAG
Acot1	TGCACGAGCGTCACTTCTT	GATACTCCAGAAGGCCACCTC
Acot3	GCACGAGCGTCACTTCAT	CGATACTCCAGAAGGCCACT
Hmox1	AACACTCTGGAGATGACACCT	TGTGAGGGATCTGGTCTTTG
Itgal	CCAGACTTTTGCTACTGGGAC	GCTTGTTCGGCAGTGATAGAG
Icam	TCCGCTACCATCACCGTGTAT	TAGCCAGCACCGTGAATGTG
Tlr1	TGTGAATGCAGTTGGTGAAGA	CATTCCTGAGGTCCCTGCTA
Ccl3	CTCCCAGCCAGGTGTCATTTT	CTTGGACCCAGGTCTCTTTGG
Rantes	GCTGCTTTGCCTACCTCTCC	TCGAGTGACAAACACGACTGC
Ifngr1	GTGGAGCTTTGACGAGCACT	TTCCCAGCATACGACAGGGT
IL-5	CTCTGTTGACAAGCAATGAGACG	TCTTCAGTATGTCTAGCCCCTG
IP10	CCAAGTGCTGCCGTCATTTTC	GGCTCGCAGGGATGATTTCAA
Mig	GGAGTTCGAGGAACCCTAGTG	GGGATTTGTAGTGGATCGTGC
Nos2	AATCTTGGAGCGAGTTGTGG	CAGGAAGTAGGTGAGGGCTTG
CD301	TGAGAAAGGCTTTAAGAACTGGG	GACCACCTGTAGTGATGTGGG
CD163	TCCACACGTCCAGAACAGTC	CCTTGGAAACAGAGACAGGC
Atrogin	ATGCACACTGGTGCAGAGAG	TGTAAGCACACAGGCAGGTC
Murf	CCACCAAACTTGTGGAGACC	CATGTTCTCAAAGCCTTGCTC
Atf4	CTGGATTCGAGGAATGTGCT	CCACCATGGCGTATTAGAGG

PCR primers for human transcripts

Primer	Forward sequence 5' → 3'	Reverse sequence 5' → 3'
β-Actin	CAAGGCCAACCGCGAGAAGAT	CCAGAGGCGTACAGGGATAGCAC
p21^{Cip1}	TGTCCGTCAGAACCCATG	TGCCTCCTCCCAACTCATC
